# Chinese Classical Music Lowers Blood Pressure and Improves Left Ventricular Hypertrophy in Spontaneously Hypertensive Rats

**DOI:** 10.3389/fphar.2022.826669

**Published:** 2022-05-02

**Authors:** Jingyuan Li, Zhi Yang, Chunmei Zhang, Yang Hu, Hongxuan Li, Meng Zhang, Peili Bu, Shuangxi Wang, Cheng Zhang, Wenjing Li

**Affiliations:** ^1^ The Key Laboratory of Cardiovascular Remodeling and Function Research, Chinese Ministry of Education, Chinese National Health Commission and Chinese Academy of Medical Sciences, The State and Shandong Province Joint Key Laboratory of Translational Cardiovascular Medicine, Department of Cardiology, Qilu Hospital, Cheeloo College of Medicine, Shandong University, Jinan, China; ^2^ Fine Arts School of Shandong University, Jinan, China

**Keywords:** music therapy, Chinese classical music, hypertension, myocardial remodelling, bisoprolol, β1 receptor, α1 receptor

## Abstract

High blood pressure (BP) plays an important role in the pathogenesis and development of cardiovascular diseases and multi-organ damages. Music has been well known to elicit emotional changes, such as anxiolytic effects. However, whether music therapy lowers BP in spontaneously hypertensive rats (SHR) and the potential mechanism remains unknown. SHRs were, respectively exposed to white noise (WN), Western classical music (WM), Chinese classical music (CCM), rock music (RM), and bisoprolol treatment. WN and WM did not lower systemic BP, but CCM and RM significantly lowered BPs in SHRs. The effects of CCM therapy on lowering systemic BPs is comparable to that of bisoprolol at the dose of low to medium. Combination of CCM treatment with bisoprolol further improved systemic BPs and myocardial hypertrophy in SHRs, compared to CCM treatment or bisoprolol alone. Furthermore, IHC and WB analysis indicated that CCM therapy inhibited the β1/cAMP/PKA and α1/PLC/PKC signalings, but didn’t alter the β2/PI3K/Akt signaling. Above all, CCM therapy lowers systemic BPs and alleviates myocardial hypertrophy in hypertensive rats, which may be caused by the inhibitions of β1/cAMP/PKA and α1/PLC/PKC signalings.

## Introduction

Hypertension is a growing public health problem and affects over 1.2 billion individuals worldwide. Hypertension, as one of the most common chronic diseases, can lead to major complications such as stroke, myocardial infarction, heart failure and chronic kidney disease, causing serious social and economic burden to the society (Flint et al., 2019; Slivnick and Lampert, 2019; Dhande et al., 2021). Hypertension is a multifactorial disease involving environmental and genetic factors together with risk-conferring behaviors. Some major risk factors have been clarified, such as genetic factors, high sodium and low potassium diet, overweight and obesity, long-term mental tension (Manosroi and Williams, 2019; Saxton et al., 2019; Lee et al., 2020; Rossitto et al., 2021). However, unlike the eating patterns and body types, mental stress is difficult to quantify. This makes it difficult to conduct epidemiological studies and prospective clinical studies on the causal relationship between stress and hypertension. This also leads to the lack of evidence for stress intervention in all current hypertension treatment guidelines.

At present, clinicians mainly treat hypertension from two aspects: non-drug and drug therapy. Non-drug therapy is the cornerstone of hypertension prevention and treatment. Currently, in the United States and Europe, the recommendations for non-pharmacological treatment of hypertension include a low-sodium and low-fat diet, moderate physical activity, weight loss, tobacco abstinence, and alcohol restriction, but none of them are related to stress reduction (Williams et al., 2018). In the China’s 2009 basic edition of hypertension treatment guidelines, the suggestions for non-drug treatment also include maintaining optimism, alleviating psychological burden, overcoming paroxia, correcting bad personality, resisting adverse social factors, psychological counseling, music therapy, self-discipline training or Qigong. It is the first time that music therapy has been mentioned in the world’s national guidelines for treating hypertension. But it does not give specific recommendations for music therapy.

In 1979, Janiszewski M published the study of music treatment of hypertension for the first time in the world, opening up a new field of music treatment for cardiovascular diseases (Janiszewski, 1979). However, for more than half a century, music therapy received little attention from the medical community. Recent studies have shown that music therapy can reduce the occurrence of anxiety and depression, relieve the clinical symptoms of Parkinson’s disease, epilepsy, Alzheimer’s disease and other diseases, promote the repair of brain injury and so on (Koshimori and Thaut, 2018; Leggieri et al., 2019). For patients with grade 1 hypertension and over the age of 50, implementation of music therapy for 2 months caused systolic blood pressure and diastolic blood pressure significantly reduced (Zanini et al., 2009). For patients with anxiety-related hypertension before and after surgery, music therapy can significantly reduce blood pressure (Allen et al., 2001).

Our recent research shows that in mice with anxiety caused by mutations in the brain-derived neurotrophic factor (BDNF) gene, music therapy reversed anxiety symptoms and increased BDNF mRNA and protein levels in several brain regions. Its molecular mechanism is that music therapy increased the BDNF receptor TrkB mRNA expression level (Li et al., 2010). These above studies suggest that music therapy for hypertension has theoretical and experimental basis. However, up to now, large sample clinical evidence of music therapy for lowering blood pressure is lacking, and the molecular mechanism of music therapy and its effect on target organ damage are unknown.

A large number of experimental and clinical evidence indicates that sympathetic excitation and activation of the RAS system play a central role in the pathogenesis of hypertension (Jackson et al., 2018; Arendse et al., 2019; Hirooka, 2020). Sympathetic nerve stimulation can increase the release of catecholamines (epinephrine and norepinephrine). Adrenergic receptors in cardiomyocytes are a class of G-protein-coupled receptors, including β1, β2 and α1 subtypes. Although it is theoretically speculated that music therapy may act by inhibiting the sympathetic nerve, the effect of music therapy on the adrenergic receptor signaling pathway in cardiomyocytes has not been reported.

Compared with medication for hypertension, music therapy has the advantages of simplicity, economy, no side effects and higher compliance, so it has a potential clinical application prospect. However, there are a number of important scientific issues that need to be addressed in this area. Firstly, previous published clinical studies on music therapy for hypertension have mostly involved small populations, and large clinical evidence is lacking. Secondly, the standardization of music therapy is uncertain. Western classical music, such as Mozart’s repertoire, was mostly used in previous foreign studies, but there were no randomized comparative studies on the antihypertensive effects of different types of Western music. No one has compared the antihypertensive effects of Chinese and Western classical music. Thirdly, the antihypertensive effect of music therapy is unclear compared with traditional antihypertensive drugs with clear efficacy. Whether music therapy and drug therapy have additive or synergistic antihypertensive effects is unknown. Furthermore, previous studies only evaluated the antihypertensive effect of music therapy, but whether music therapy can improve the target organ damage of hypertension is unclear. And no studies have been reported on the effects of music therapy on sympathetic nerve activity, expression of adrenergic α1, β1 and β2 receptor subtypes, expression of receptor-coupled proteins GQ, GS and GI, and intracellular PKC, PKA and PI3K signaling pathways.

In this study, we compared four types of music therapy, including Western classical music (WM), rock music (RM), Chinese classical music (CCM) and noise on systemic BPs in rats. Our results indicated that CCM is more effective to lower BP and improves cardiac function in spontaneously hypertensive rats (SHR). And the main mechanism of CCM lowering blood pressure is the down-regulation of β1/cAMP/PKA and α1/PLC/PKC signaling pathways.

## Materials and Methods

### Animals

Male Wistar-Kyoto (WKY) rats and spontaneously hypertensive rats (SHR), 8–12 weeks of age, 200–250 g, were obtained from the Beijing Huafukang Company (Beijing, China). Rats were housed in temperature-controlled cages with a 12-h light-dark cycle and given free access to water and food. All animal protocols were approved by the Institutional Animal Care and Use Committee of Cheeloo College of Medicine, Shandong University. All relevant ethical regulations were adhered to.

### Music and Drug Treatment

As described by us (Li et al., 2010), for music treatment, rats were exposed to music daily for 10 weeks. Music or noise was played from 19:00 to 7:00 for 12 h during the active time of rats. The distance between rat cage and sound box was kept in 1 m. All experiments were carried out in a quiet environment to avoid other voice added. The sound level of the control group was under 40 dB (ambient noise). Music of 50–60 dB was played on a CD player with a frequency of 300–10,000 Hz in the home cages. Bisoprolol (from Sigma Corp.,United States) was given by oral administration at 2.5, 5 or 10 mg/kg/day for 10 weeks.

### Music Selection

For white noise, rats were treated with irregular noises of 50–100 Hz generated by a high-frequency noise generator. For Chinese classical music, we selected classical instrumental music from the Qin and Han dynasties to the Sui and Tang Dynasties, including guqin music, pipa music, silk and bamboo music and guchui music written in pentatonic mode of the ancient scale. The music reflect the characteristics of more rhymes and less tones. The tone is simple and passionate, the rhythm is natural and free, the melody is euphemistic and delicate, and the meaning of the song is meaningful. For Western classical music, we selected the classical works of some composers from the Baroque, classical and romantic periods. The music is characterized by varied and dynamic rhythms, multi-voice harmonic thinking to construct the musical texture, and melody balance and symmetry. Selected repertoire includes of piano music and symphony music, such as Bach piano with strict and balanced style, Handel chorus and Concerto with bright, cheerful and comely style, Beethoven sonata with balanced tonality and elegant style, Haydn symphonies with drama and philosophy and Mozart and Schumann piano pieces with pure beauty. For rock music, we chose songs from the 1980s that are a mixture of singing and loud electroacoustic instruments, characterized by a simple musical vocabulary, strong rhythm, shock and harsh sound effects.

### Blood Pressure Measurement

Blood pressure was determined by invasive radio telemetry methods as described previously (Yan et al., 2020; Zhang et al., 2022). Briefly, rats were implanted with a TA11PA-C10 radio telemetry transmitter (Data Sciences, Laurel, Md) for 24-h recording of arterial pressure and heart rate with a radio telemetry data-acquisition program (Dataquest ART 3.1, Data Sciences). Hemodynamic measurements were sampled for 10 s every 10 min for the 3-week duration. Data were reported as 24-h average.

### Echocardiography

The heart function and dimension parameters were measured using a standard protocol by transthoracic parasternal echocardiography using the VEVO770 imaging system (Visual Sonics, Toronto, ON, Canada). LV parameters, including left ventricular ejection fraction (LVEF), fractional shortening (FS), the interventricular septum (IVSd) and left ventricular posterior wall thickness (LVPWd), were measured in M-mode via the long/short axis view.

### Histopathology and Immunohistochemistry

As described previously (Zhang M. et al., 2021), the heart tissues were isolated and fixed in 4% paraformaldehyde and maintained at 4°C until use. The fixed tissues were dehydrated and processed for paraffin embedding. 5-μm sections were stained with hematoxylin and eosin (H&E) and immunohistochemistry assays. The deparaffinized, rehydrated section from hearts were microwaved in citrate buffer for antigen retrieval. Sections were incubated in endogenous peroxidase and protein block buffer, and then with primary antibodies indicated overnight at 4°C. Slides were rinsed with washing buffer and incubated with labelled polymer-horseradish peroxidase-antimouse/antirabbit antibodies followed by DAB + chromogen detection. After final washes, sections were counterstained with hematoxylin. All positive stainings were confirmed by ensuring that no staining occurred under the same conditions with the use of non-immune rabbit or mouse control IgG. Semi-quantitative analysis of the integrated optical density (IOD) was analyzed by software of Image-Pro Plus 5.1. The IOD is expressed as positive area × intensity.

### Western Blot Analysis

Total proteins were extracted from rat hearts and equal amounts of protein were electrophoretically separated and then transferred to PVDF membranes. The membranes were incubated overnight at 4°C with the corresponding primary antibodies. Anti-rabbit IgG and anti-mouse IgG antibodies were used as secondary antibodies. The membranes were developed using Immobilon Western Detection Reagents (Millipore, Billerica, MA, United States). The intensity of bands was measured by using ImageJ. All experiments were repeated at least three times and mean values were derived. Primary antibodies against α1 receptor, β1 receptor, β2 receptor, PI3K, Akt, PLC, cAMP, PKA, and PKC and secondary antibodies were obtained from Cell Signaling technology (CST Corp.,United States).

### Statistical Analysis

All quantitative results are expressed as mean ± SEM. After testing for normality and equality of variance, continuous data were compared by unpaired Student’s t-test or one-way analysis of variance (ANOVA). Bonferroni corrections were applied to multiple tests. Statistical analysis was conducted using IBM SPSS statistics 20.0 (IBM Corp., Armonk, NY, United States) and *p* < 0.05 were considered statistically significant.

## Results

### Meta-Analysis Indicates That Music Therapy Lowers BP in Hypertensive Patients

**FIGURE 1:**
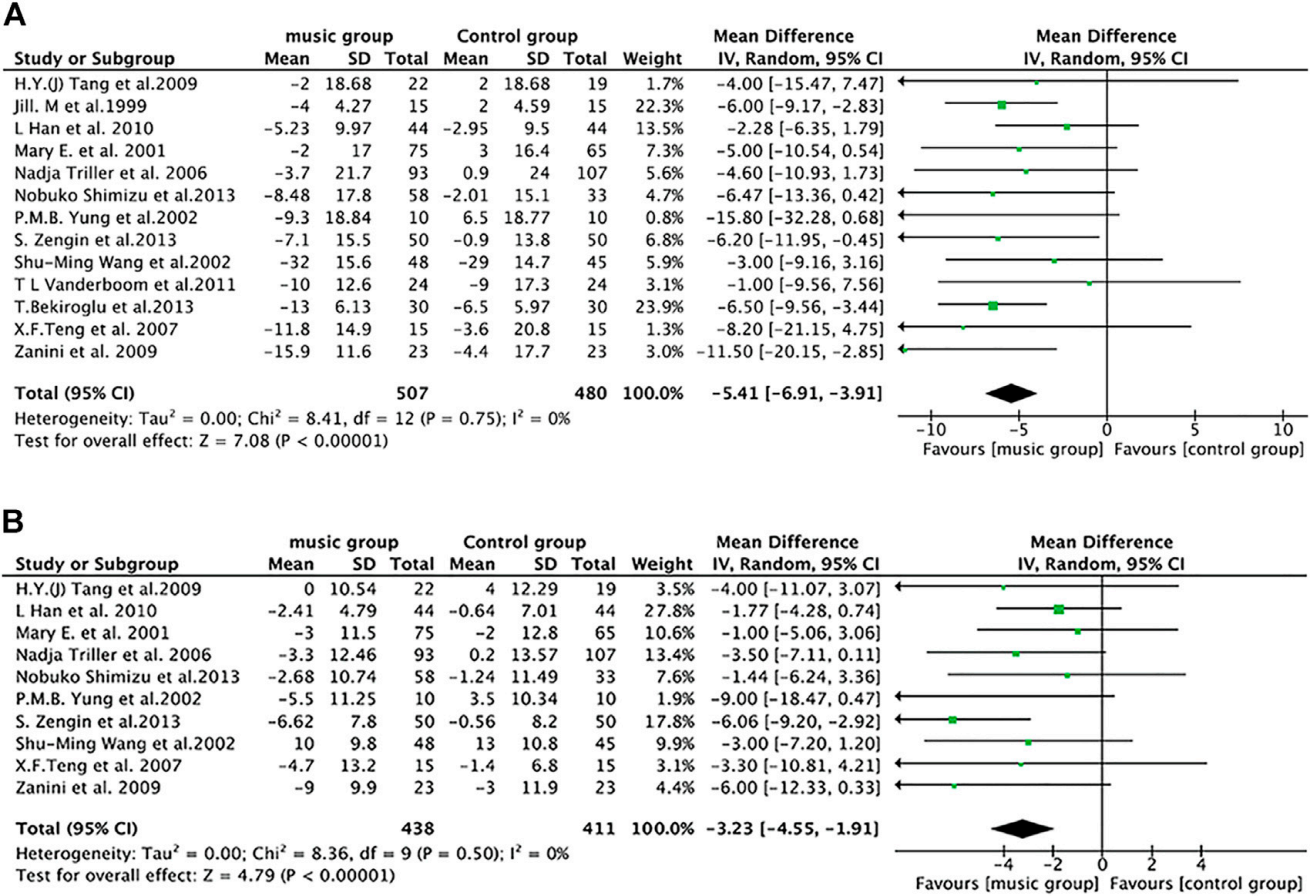
Meta-analysis of music therapy on blood pressure (BP). **(A)** Systolic BP. **(B)** Diastolic BP. Odds ratios (95% CI) for music vs. control.

We first performed a meta-analysis to find the association between music therapy and hypertension. In all the included studies, 987 patients from 13 trials provided data of systolic BP change from the baseline to the end-point of treatment period. Figure 1A shows the forest plot of different lowering systolic BP efficacy between music group and control group. The final merged results show that music therapy is significantly effective in lowering systolic BP (pooled effect size [95%CI]: −5.41 [−6.91, −3.91] (*p* < 0.00001); Heterogeneity: Tau^2^ = 0.00; Chi^2^ = 8.41, df = 12 (*p* = 0.75); I^2^ = 0%. The heterogeneity doesn’t exist).

The meta-analysis of diastolic BP reductions between the music group and control group also showed a statistically meaningful result. As shown in Figure 1B, among 849 subjects from 10 trials, patients who listen to music possess lower diastolic BP compared to that of control group (pooled effect size [95%CI]: −3.23 [−4.55, −1.91]; Heterogeneity:Tau^2^ = 0.00; Chi^2^ = 8.36, df = 9 (*p* = 0.5); I^2^ = 0%. The heterogeneity doesn’t exist). Taking all these data together, it demonstrates that music therapy lowers BP in hypertensive patients.

### Both CCM and RM Therapies Lower Systemic BPs in Spontaneously Hypertensive Rats

**FIGURE 2:**
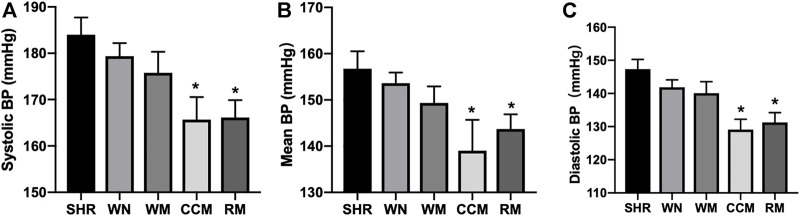
Chinese classical Music (CCM) therapy lowers systemic BPs in spontaneously hypertensive rats (SHRs). Male SHRs at the age of 8–12 weeks old fed with normal diet were exposure to indicated music therapy. WN, white noise; WM, Western classical music; CCM, Chinese classical music; RM, Rock music. **(A)** Systolic BP, **(B)** mean BP and **(C)** diastolic BP were analyzed. Quantitative results are expressed as mean ± SEM. N is 10–15 in each group. *p < 0.05 vs. SHR mice.

The results of meta-analysis from clinical observations were further confirmed by our experimental investigations in animals. We investigated the effects of music therapy on hypertensive rats by exposing SHR to WN, WM, CCM and RM for 10 weeks. Systemic BPs, including systolic pressure, diastolic pressure and mean arterial pressure, were measured by radio telemetry method. As indicated in Figures 2A–C, compared to systemic BPs in control SHRs (184.0 ± 3.7 mmHg for systolic BP; 147.4 ± 3.3 mmHg for diastolic BP; 156.8 ± 3.8 for mean BP), WN did not lower systemic BP (179.4 ± 2.8 mmHg for systolic BP; 141.9 ± 2.2 mmHg for diastolic BP; 153.6 ± 2.3 mmHg for mean BP) in SHRs. WM therapy did not reduce systemic BPs (175.8 ± 4.5 mmHg for systolic BP; 140.1 ± 3.5 mmHg for diastolic BP; 149.4 ± 3.6 mmHg for mean BP). Both CCM therapy and RM therapy significantly deceased the systemic BPs in SHRs (CCM: 165.7 ± 4.9 mmHg for systolic BP; 129.0 ± 3.1 mmHg for diastolic BP; 139.0 ± 6.7 mmHg for mean BP; RM: 166.2 ± 3.7 mmHg for systolic BP; 131.3 ± 3.0 mmHg for diastolic BP; 143.7 ± 3.2 mmHg for mean BP). There is no significant difference between the CCM and RM groups.

### CCM Therapy on Hypertension is Comparable to Bisoprolol at the Dose of Low to Medium in Hypertensive Rats

**FIGURE 3:**
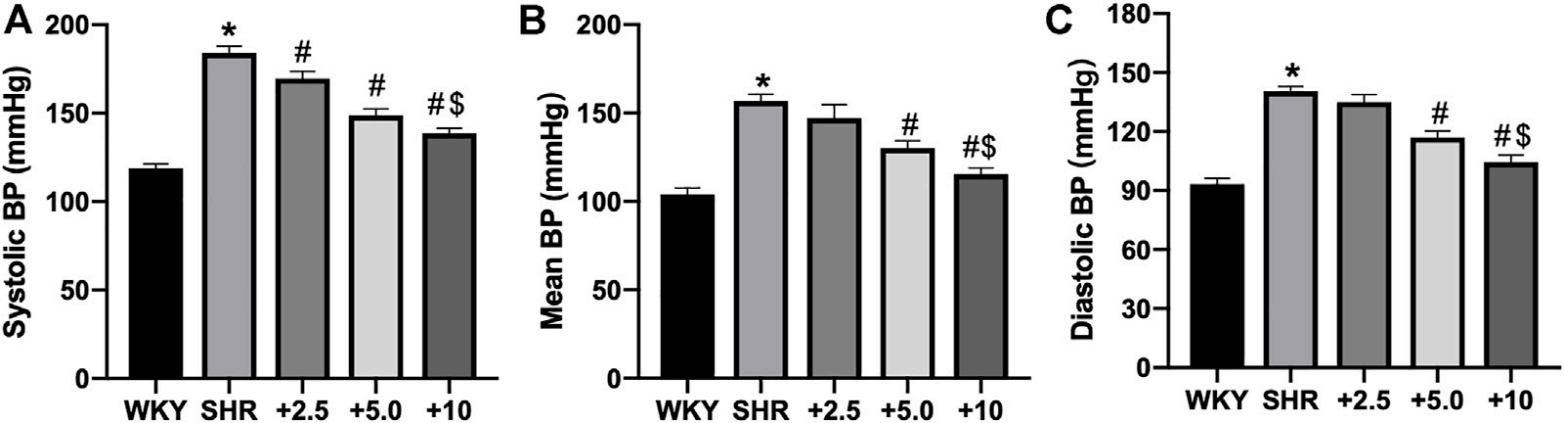
Bisoprolol administration dose-dependently reduces systemic BPs in SHRs. Male SHRs at the age of 8–12 weeks old fed with normal diet received bisoprolol administration (2.5, 5.0, 10 mg/kg/day) in drinking water for 10 weeks. BP was measured by radio telemetry, as described in Materials and Methods. Wistar rats served as control. **(A)** Systolic BP, **(B)** mean BP and **(C)** diastolic BP were analyzed. Quantitative results are expressed as mean ± SEM. N is 10–15 in each group. *p < 0.05 vs. WKY rats. #*p* < 0.05 vs. SHRs. $*p* < 0.05 vs. 5.0 mg/kg/day bisoprolol group.

We next compared the effects of music therapy with drug treatment on hypertension. Bisoprolol, a highly selective β1 receptor antagonist, is served as a drug to prevent hypertension (Sabidó et al., 2018). WKY rats were used as normal control. As shown in Figures 3A–C, bisoprolol administration does-dependently lowered systemic BPs, compared to SHRs. However, bisoprolol at high-dose (10 mg/kg/day) still did not decrease systemic BPs to the normal level (138.6 ± 2.8 vs. 118.7 ± 2.7 mmHg for systolic BP, *p* < 0.05; 115.4 ± 3.5 vs. 103.8 ± 3.8 mmHg for diastolic BP; 104.5 ± 3.5 vs. 93.4 ± 2.8 mmHg for mean BP, *p* < 0.05). The effects of CCM therapy on systemic BPs were comparable to the effects of bisoprolol at the low-medium dose in spontaneous hypertensive rats.

### CCM Therapy Amplifies the Lower-BP Effects of Bisoprolol in Rats

**FIGURE 4:**
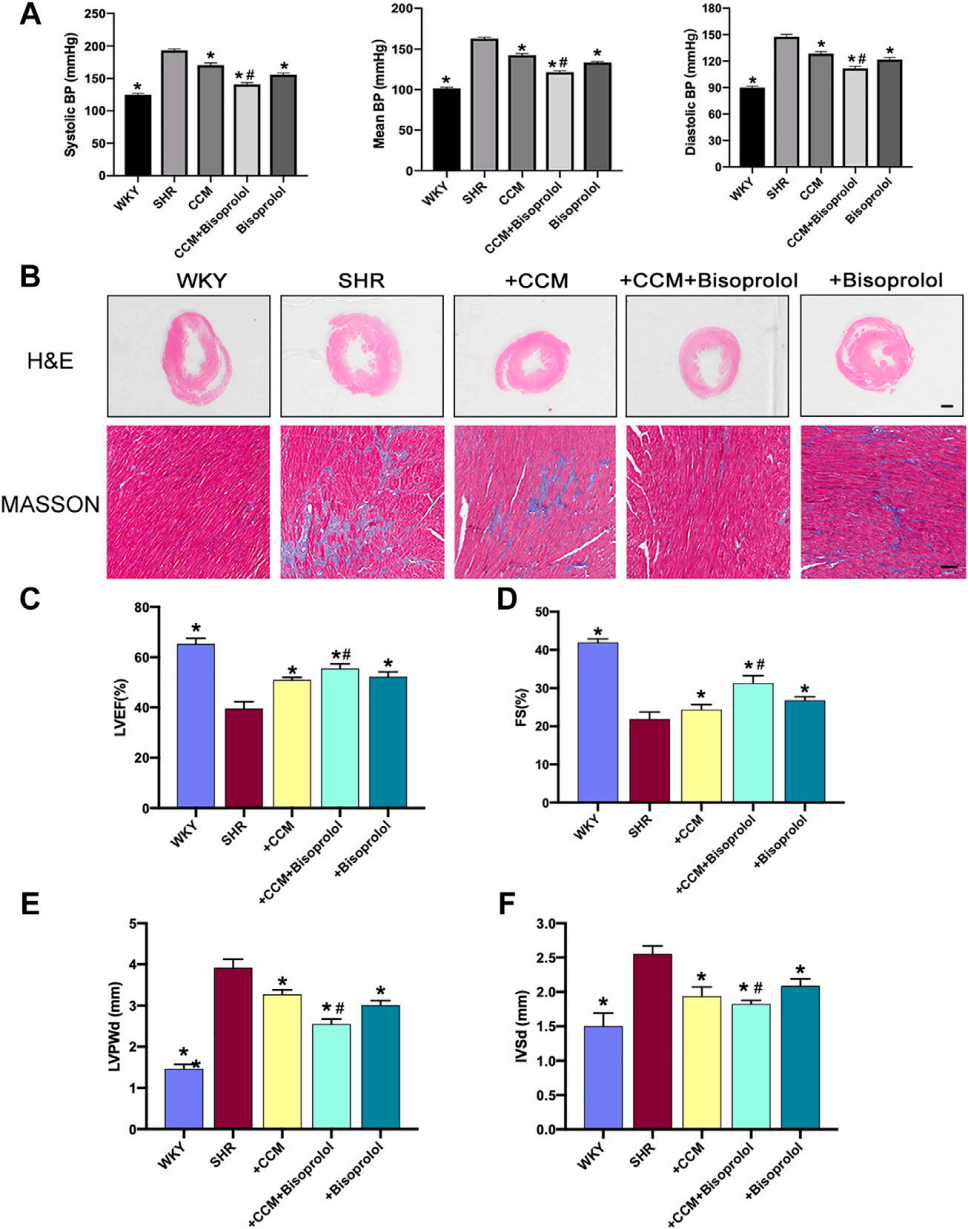
CCM therapy promotes bisoprolol-produced protective effects on hypertension and myocardial remodeling in SHRs. Male SHRs at the age of 8–12 weeks old fed with normal diet received bisoprolol administration (5 mg/kg/day) or plus CCM therapy for 10 weeks. BP was measured by radio telemetry, as described in Materials and Methods. **(A)** Systolic BP, mean BP and diastolic BP; **(B)** HE staining, scale bar: 2mm; MASSON staining, scale bar:100 μm; **(C–F)** Left ventricular ejection fraction (LVEF), Fractional shortening (FS), thickness of the left ventricular diastolic posterior wall (LVPWd), thickness of interventricular septum (IVSd). Each experiment was repeated three times. Quantitative results are expressed as mean ± SEM. N is 10–15 in each group. *p < 0.05 vs. SHRs. #*p* < 0.05 vs. SHRs plus bisoprolol.

Considered that high-dose bisoprolol did not reduce BP to normal level and CCM therapy lowered BP, which is equal to bisoprolol at low or medium dose, we hypothesized that CCM therapy can be considered as supplementary treatment for bisoprolol to lower BP. To test this hypothesis, SHRs received therapy of medium-dose bisoprolol or CCM or bisoprolol plus CCM. As shown in Figure 4A, compared to bisoprolol, combination of CCM further decreased systemic BPs in bisoprolol-treated SHRs (140.7 ± 2.9 vs. 156.0 ± 2.6 mmHg for systolic BP, *p* < 0.05; 111.8 ± 2.3 vs. 121.8 ± 2.4 mmHg for diastolic BP, *p* < 0.05; 121.5 ± 1.7 vs. 133.3 ± 1.2 mmHg for mean BP). These data demonstrate that bisoprolol plus CCM therapy is more effective than bisoprolol alone.

### CCM Therapy Increases Bisoprolol-Induced Improvement of Myocardial Remodeling in Hypertensive Rats

Hypertension is a critical risk factor of left ventricle hypertrophy (Yildiz et al., 2020). We next examined the effects of CCM therapy on bisoprolol-induced protective effects in myocardial remodeling. Differences in heart weight and the ratio of heart weight to body weight were statistically significant among the five groups (Table 1). Meanwhile, masson staining showed that the cardiac fibrosis was improved in the combination treatment group, compared to CCM or Bisoprolol treatment alone (Figure 4B). The echocardiography results showed that therapy of bisoprolol plus CCM further elevated the left ventricular ejection fraction (LVEF) and fractional shortening (FS), decreased the thickness of left ventricle posterior wall (LVPWd) and interventricular septum (IVSd) in SHRs, indicating that bisoprolol plus CCM therapy is more effective than bisoprolol alone on improvement of myocardial remodeling (Figures 4C–F).

**TABLE 1:** Characteristics of the five groups of rats after 10 weeks of treatment.

Parameters	WKY	SHR			
		Control	+CCM	+CCM + Bisoprolol	+Bisoprolol
Body weight (BW, g)	313.16 ± 7.98	318.36 ± 8.24	317.45 ± 7.73	315.42 ± 5.95	313.95 ± 7.14
Heart weight (HW, mg)	1098.44 ± 37.39	1361.53 ± 46.44^a^	1247.05 ± 18.63^b^	1159.65 ± 22.22^bc^	1198.90 ± 31.55^b^
HW/BW (mg/g)	3.51 ± 0.08	4.29 ± 0.17^a^	3.93 ± 0.09^b^	3.69 ± 0.12^bc^	3.82 ± 0.14^b^

^a^ p < 0.05 vs. WKY.

^b^ p < 0.05 vs. SHRs, Control.

^c^ p < 0.05 vs. SHRs + Bisoprolol.

### CCM Therapy Decreases β1/cAMP/PKA Signaling

**FIGURE 5:**
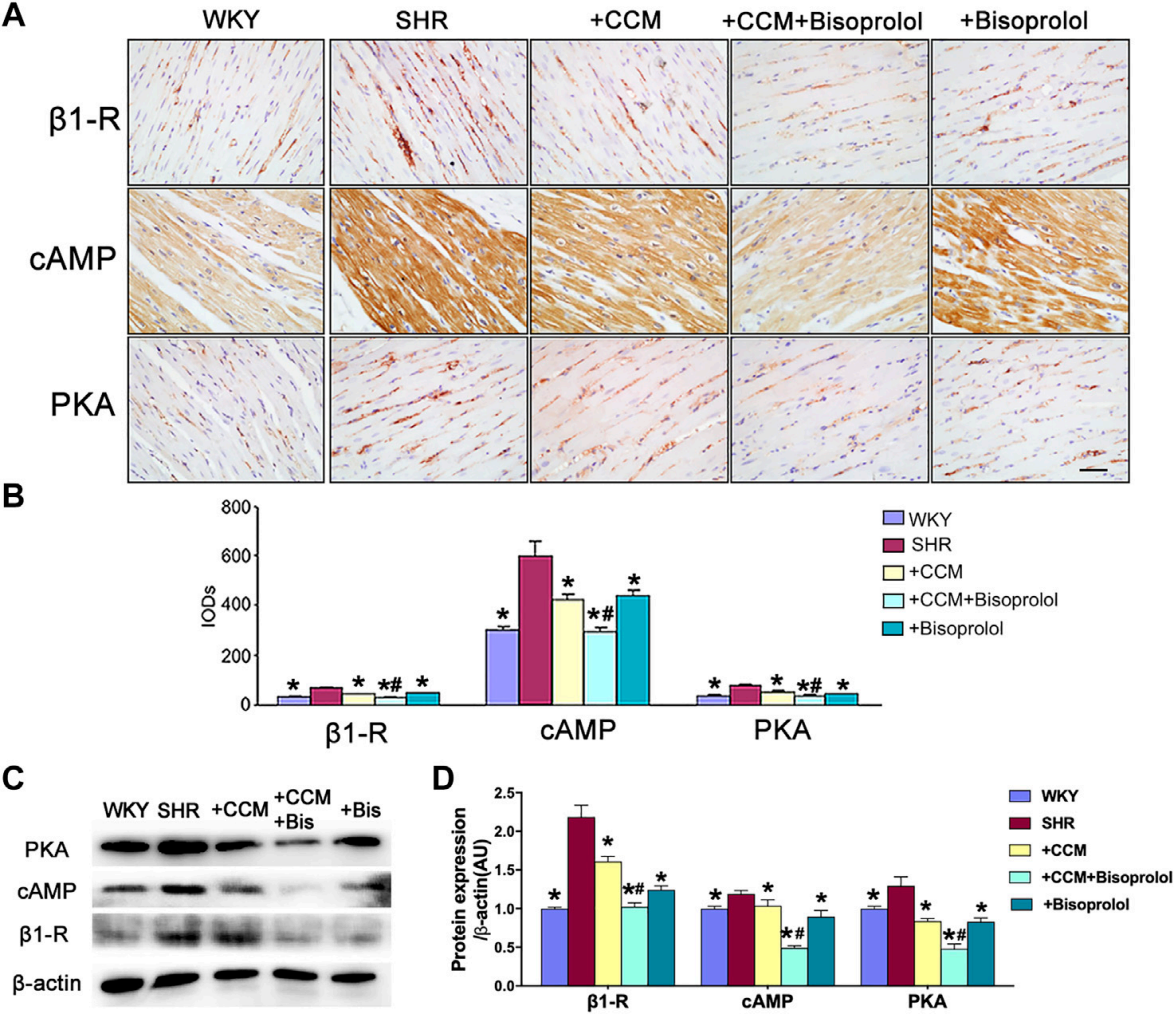
CCM therapy inhibits β1/cAMP/PKA signaling in SHRs hearts. Male SHRs at the age of 8–12 weeks old fed with normal diet received bisoprolol administration (5 mg/kg/day) or plus CCM therapy for 10 weeks. Wistar rats served as control. **(A,B)** Immunohistochemistry and quantitative analysis of β1/cAMP/PKA signaling in hearts. scale bar:200 μm. **(C,D)** Western blot and quantitative analysis of β1/cAMP/PKA signaling in hearts. Quantitative results are expressed as mean ± SEM. N is 10–15 in each group. *p < 0.05 vs. SHRs. #*p* < 0.05 vs. SHRs plus bisoprolol.

As described above, CCM therapy produced similar effects of bisoprolol at low-medium dose. We hypothesized that CCM therapy also blocks β1-receptor to lower BP and improve myocardial remodeling in SHRs treated with bisoprolol. To test this concept, we measured β1-receptor-depndent cAMP/PKA signaling in hearts. As showed in Figures 5A–D, immunohistochemistry and western blot analysis indicated that both CCM therapy and bisoprolol reduced β1/cAMP/PKA signaling in hearts of SHRs. However, combination of CCM with bisoprolol further decreased β1/cAMP/PKA signaling, suggesting that CCM therapy may inhibit β1/cAMP/PKA signaling to increase the protective effects of bisoprolol on SHRs.

### CCM Therapy Reduces α1/PLC/PKC Without Effects on β2/PI3K/Akt Signaling

**FIGURE 6:**
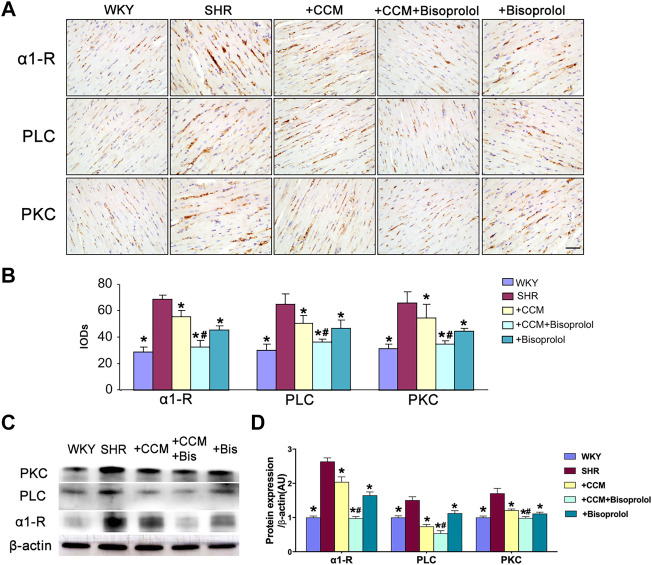
CCM therapy reduces the α1/PLC/PKC signaling in SHRs hearts. Male SHRs at the age of 8–12 weeks old fed with normal diet received bisoprolol administration (5 mg/kg/day) or plus CCM therapy for 10 weeks. **(A,B)** Immunohistochemistry and quantitative analysis of α1/PLC/PKC signaling in hearts. scale bar:200 μm. **(C,D)** Western blot and quantitative analysis of α1/PLC/PKC signaling in hearts. Quantitative results are expressed as mean ± SEM. N is 10–15 in each group. *p < 0.05 vs. SHRs. #*p* < 0.05 vs. SHRs plus bisoprolol.

**FIGURE 7:**
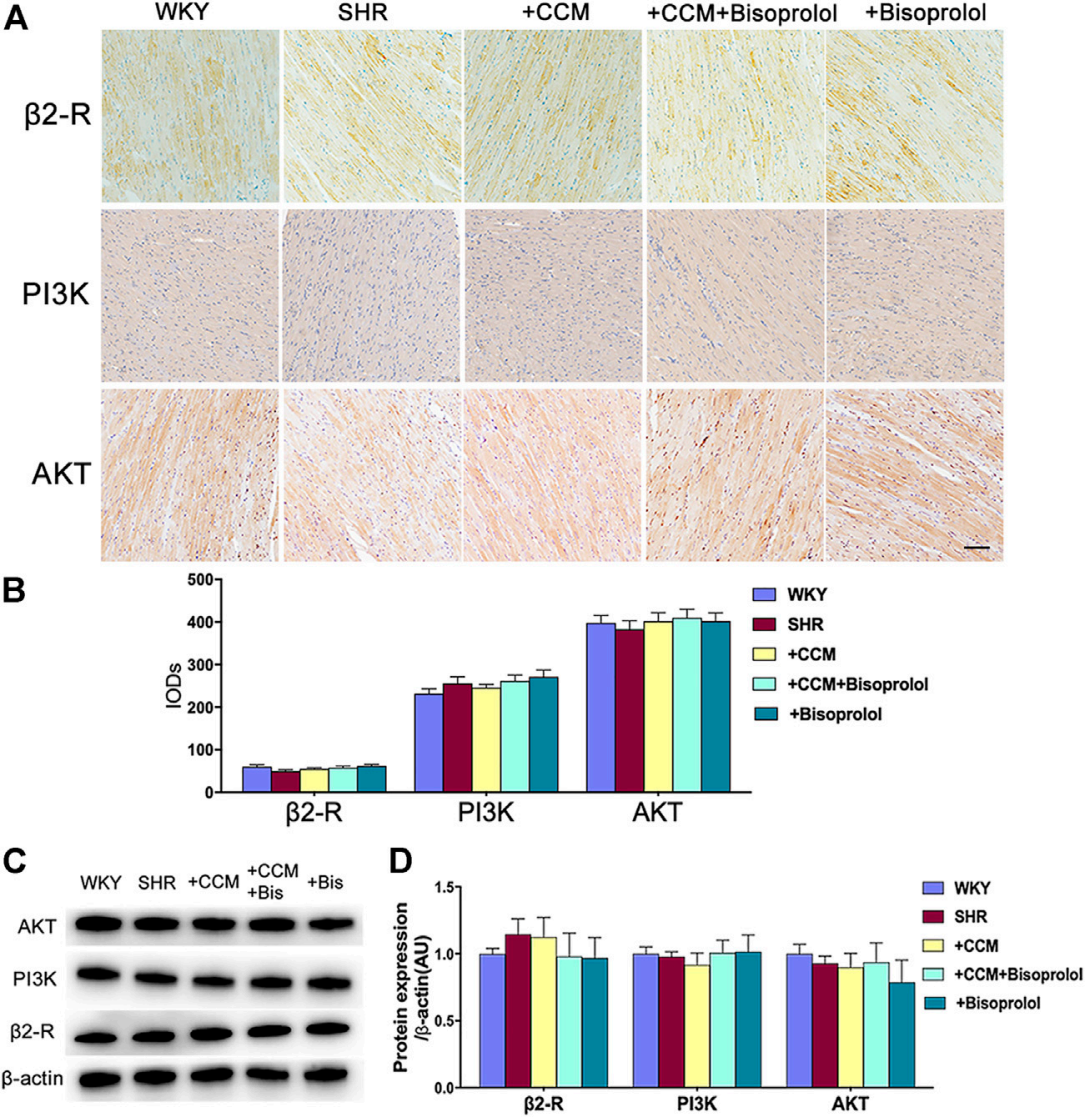
CCM therapy does not alter β2/PI3K/Akt signaling in SHRs hearts. Male SHRs at the age of 8–12 weeks old fed with normal diet received bisoprolol administration (5 mg/kg/day) or plus CCM therapy for 10 weeks. **(A,B)** Immunohistochemistry and quantitative analysis of β2/PI3K/Akt signaling in hearts. scale bar:100 μm. **(C,D)** Western blot and quantitative analysis of β2/PI3K/Akt signaling in hearts. Quantitative results are expressed as mean ± SEM. N is 10–15 in each group.

Besides β1/cAMP/PKA signaling, either α1/PLC/PKC signaling or β2/PI3K/Akt signaling is also involved in myocardial remodeling (Nakamura and Sadoshima, 2018; Zhang J. et al., 2021). Thus, we detected β2/PI3K/Akt or α1/PLC/PKC signaling in the hearts of SHRs. As depicted in Figures 6A–D, both CCM therapy and bisoprolol reduced α1/PLC/PKC signaling in hearts of SHRs. As expected, combination of CCM with bisoprolol further decreased α1/PLC/PKC signaling. Interestingly, CCM therapy plus bisoprolol did not alter the level of β2/PI3K/Akt signaling, compared to CCM therapy or bisoprolol (Figures 7A–D). These data indicate that α1/PLC/PKC but not β2/PI3K/Akt signaling is involved in the effects of CCM therapy.

## Discussion

Hypertension has become one of the most common chronic diseases worldwide and is a major risk factor for atherosclerotic cardiovascular disease. Advanced hypertension can lead to stroke, heart failure, myocardial infarction, renal failure, aortic dissection and other serious complications. Therefore, early prevention, early detection and early treatment are extremely important. The mass prevention and treatment of hypertension has become a major task faced by the health prevention and medical institutions. Interventions for patients with hypertension can be divided into non-pharmacological and pharmacological treatments, and non-pharmacological treatment should be implemented first except for acute and severe hypertension. In the China’s 2009 basic edition of hypertension treatment guidelines, it was the first time that music therapy has been mentioned in the guidelines for the treatment of hypertension in any country in the world, which is a major innovation. But it does not give advice on how to conduct music therapy. Therefore, it has become an important topic in the field of hypertension treatment to establish clinical and experimental evidence of blood pressure reduction by music therapy, compare the relative and synergistic effects of different types of music and traditional anti-hypertensive drugs, and study the molecular mechanism of blood pressure reduction by music therapy.

Since Sear HG first published his paper on music therapy in 1946 (Sear, 1946), music therapy has been used as an adjuvant therapy for a small number of neuropsychiatric disorders. In 1979, Janiszewski M first published the study of music in the treatment of hypertension, which opened up a new field of music in the treatment of cardiovascular diseases. Studies have shown that music therapy can slow the heart rate, relieve anxiety and improve clinical symptoms such as headaches, dizziness and chest pain. Therefore, music therapy has been used in the field of mental illness and rehabilitation medicine, aiming to improve the mental and physical state of patients (Wang and Agius, 2018; Monsalve-Duarte et al., 2021). However, there is a lack of large-sample clinical evidence on whether music therapy is effective in reducing blood pressure. In this study, we adopted a new retrieval strategy related to music and blood pressure, rather than retrieving the literature on music in the treatment of hypertension, thus obtaining a larger sample size and significantly enhancing the efficacy of the summary analysis. Our results showed that in a randomized clinical trial of 1,063 patients, the music treatment group reduced systolic blood pressure by 5.41 mmHg and diastolic blood pressure by 3.23 mmHg, which were statistically significant. It should be noted that of the 12 studies that met the inclusion criteria for this pooled analysis, only 5 were on hypertension. The mean reduction in systolic and diastolic blood pressure by music therapy was 9.2 and 4.8 mmHg, respectively. These results suggest that music therapy may reduce blood pressure in patients with hypertension more than in patients without hypertension. On the other hand, music therapy can reduce blood pressure even in patients with anxiety disorders with normal blood pressure. A recently published pooled analysis of the results of 10 clinical trials failed to draw a statistically significant conclusion due to the lack of complete data on the control group (Soares do Amaral et al., 2016). Another pooled analysis published in the same year, which included only three small clinical studies, found that music therapy reduced systolic blood pressure in patients with hypertension, but had no effect on diastolic blood pressure (Kunikullaya et al., 2015). The pooled analysis of this study provides the first large sample of clinical evidence that music therapy reduces systolic and diastolic blood pressure in patients with hypertension and anxiety.

In this study, we investigated for the first time the effects of three different types of music, including Western classical music (WM), rock music (RM), and Chinese classical music (CCM), on blood pressure in Wistar rats with normal blood pressure and spontaneously hypertensive SHR rats. In this study, for the first time, we found that different types of music had no effect on blood pressure in Wistar rats with normal blood pressure, but for SHR rats, noise stimulation had no significant effect on blood pressure (not shown in data). Western classical music showed a trend of improvement but no significance. Chinese classical music and rock music could significantly reduce blood pressure.

Although music therapy has been used in the treatment of hypertension, there are many problems in the studies published in the literature. Most of the studies are non-randomized, and the results are affected by a variety of confounding factors, such as hypertensive course, hypertension stage, mood swings, medication, cultural background, music preference, etc. Music therapy is not standard in some aspects, such as music types, different choice of songs, different length of treatment. And the research sample is usually small, dozens of cases. In the past, the selection of music for music therapy was relatively simple, and western classical music was basically chosen. However, the selection of music by different ethnic groups may vary with the cultural background of different ethnic groups, resulting in different antihypertensive effects. Above all, the previous conclusions on the treatment of hypertension by western classical music in a small sample of western populations are not necessarily applicable to all ethnic groups in the world. Since there is no difference in ethnic, historical and cultural background in rats, the rat experiment should be able to prove the effect of different music therapy on blood pressure more objectively in theory. Surprisingly, in the SHR rat model of this study, western classical music did not exert the hypotensive effects reported in previous studies in western populations. This may be due to two reasons. Firstly, the influence of western classical music on blood pressure in western populations is closely related to the historical, cultural and educational background of western populations. Secondly, in previous studies on the treatment of western classical music, the subjects were mostly patients with grade I hypertension, and the blood pressure of SHR rats was as high as 180/140 mmHg. Therefore, western classical music may not have an obvious effect on hypertension above grade III.

Previously published studies on music in the treatment of hypertension have not compared the relative and synergistic effects of music therapy with traditional antihypertensive drugs, and this study is the first to compare the two therapies. A 2015 study comparing music therapy with lifestyle improvements versus lifestyle improvements alone found that music therapy significantly reduced systolic and diastolic blood pressure in patients with hypertension and prehypertension (Kunikullaya et al., 2015). At present, there is a lack of comparative studies between music therapy and drug therapy in the literature, so the value of music therapy relative to traditional drug therapy is unknown. Bisoprolol is a highly selective β1 receptor blocker that inhibits sympathetic activation and has been widely used in the treatment of hypertensive patients (DiNicolantonio et al., 2013; Lin et al., 2013). Since previous studies suggested that music therapy is likely to play its role by regulating autonomic nerve balance, we selected the Chinese classical music therapy group with the most obvious antihypertensive effect and compared the antihypertensive effect with the moderate dose of bisoprolol group. The results showed that there was no significant difference in the antihypertensive effect between the two groups, suggesting that the antihypertensive effect of Chinese classical music was similar to that of medium doses of bisoprolol. In addition, our results show that, even if the high dose of the bisoprolol, nor will the blood pressure down to normal levels, so we discussed the synergistic effect of Chinese classical music therapy and middle dose of bisoprolol. We found that the effects of drugs and music combination is superior to pure drugs or pure music therapy, hints of Chinese classical music therapy can be used as a collaborative approach of drug treatment. These results suggest that if the antihypertensive effect of medium dose bisoprolol is not ideal, music therapy can be added. It can not only increase the antihypertensive effect, but also reduce the dosage of bisoprolol, avoiding drug side effects and reducing the economic burden, thus generating important social and economic benefits.

Increased hemodynamic load in high blood pressure condition cause left ventricular hypertrophy, which can lead to left ventricular ischemia and abnormal left ventricular diastolic function, and eventually the development of congestive heart failure. Therefore, left ventricular hypertrophy is an important marker of hypertensive target organ damage (Nwabuo and Vasan, 2020). Previous published studies have only evaluated the hypotensive effects of music therapy, but whether music therapy can improve myocardial remodeling in hypertension remains unclear. In this study, left ventricular wall thickness measured by ultrasound was used as an index to evaluate myocardial remodeling. It was found that Chinese classical music and medium dose bisoprolol significantly reduced left ventricular hypertrophy, and the two therapies had similar inhibitory effects on myocardial remodeling. We also observed the synergistic effect in inhibiting myocardial remodeling of Chinese classical music and bisoprolol. We found that compared with the pure music therapy or medication, combination of these two therapies can significantly reduce the left ventricular wall thickness. It suggested compared with pure drug therapy, music and drug combination therapy can effectively improve myocardial remodeling.

A large number of previous studies have proved that the activation of the sympathetic nerve plays a central role in the pathogenesis of hypertension, and the adrenergic receptors located on the membrane of different organs are the key link to mediate the function of the sympathetic nerve. Adrenergic receptor is G protein coupled receptors to accept catecholamine stimulation, which can be divided into receptor alpha and beta receptors. The adrenergic receptor in cardiomyocytes includes beta 1, 2 and alpha 1 three subtypes. The downstream of beta 1 receptor is cAMP/PKA signaling pathways, beta 2 receptor downstream is PI3K/Akt signaling pathway, and alpha 1 receptor downstream is PLC/PKC pathways. Previous studies on the treatment of hypertension by music have only observed the efficacy of antihypertensive therapy, but not the molecular mechanism. Therefore, the molecular mechanism of music in the treatment of hypertension is still unclear. In our study, we found that the expression of β1/cAMP/PKA and α1/PLC/PKC in SHR group were significantly increased compared with Wistar rats. Compared with the SHR control group, the expression levels of β1/cAMP/PKA and α1/PLC/PKC were significantly decreased in both the Chinese classical music treatment group and the medium dose bisoprolol treatment group, while the expression levels of β1/cAMP/PKA and α1/PLC/PKC were further significantly decreased in the Chinese classical music and medium dose bisoprolol combined treatment group. These results suggest that Chinese classical music therapy can down-regulate the expression levels of β1/cAMP/PKA and α1/PLC/PKC signaling, and Chinese classical music combined with and medium dose bisoprolol has synergistic effect on the inhibition of β1/cAMP/PKA and α1/PLC/PKC signaling pathways, which may explain the synergistic effect of music and drug therapy on the antihypertensive effect. On the other hand, the expression of β2/PI3K/Akt signaling pathway was not significantly affected by either Chinese classical music alone or medium dose bisoprolol, or music combined with drugs. Unfortunately, in this experiment, we only detected the expression level of PKA/PKC, but did not detect its phosphorylation level and activity. We will continue to explore the activation level and downstream pathway expression changes of PKA/PKC in future studies, so as to further reveal the specific mechanism of music therapy. These results indicate that the main mechanism of Chinese classical music lowering blood pressure is the down-regulation of β1/cAMP/PKA and α1/PLC/PKC signaling pathways. This is the first time to elucidate the molecular mechanism of music therapy lowering blood pressure, and point out the direction for further research of music therapy.

The results of this study have important clinical significance in the treatment of hypertension complicated with respiratory diseases. It is known that the α1 receptor is mainly distributed in vascular smooth muscle, the β1 receptor is mainly distributed in cardiac muscle, and the β2 receptor is distributed in both vascular and bronchial smooth muscle (O-Uchi and Lopes, 2010; Chen et al., 2010). As a result, patients with a combination of respiratory disease and hypertension have poor tolerance to non-selective beta blockers. In these patients, clinicians often use highly selective β1 blockers such as bisoprolol as antihypertensive therapy, but even highly selective β1 blockers are limited if respiratory disease is more severe. This study confirmed that the molecular mechanisms of Chinese classical music in the treatment of hypertension and inhibition of myocardial remodeling all come from the downregulation of β1/cAMP/PKA and α1/PLC/PKC signaling pathway, and does not affect the bronchial β2/PI3K/Akt signaling pathway. Therefore, Chinese classical music may be a priority for patients with severe respiratory diseases and hypertension in the future.

Taken together, the pooled analysis of this study provides the first large sample of clinical evidence that music therapy reduces systolic and diastolic blood pressure in patients with hypertension and anxiety. The basic experiment of this study confirmed that Chinese classical music therapy reduced blood pressure and inhibited left ventricular hypertrophy in SHR rats by inhibiting the β1/cAMP/PKA and α1/PLC/PKC signaling pathways. The therapeutic effect was similar to that of medium dose bisoprolol, and the combination of Chinese classical music and medium dose bisoprolol had a synergistic effect on lowering blood pressure and inhibiting left ventricular remodeling. The results of our study suggest that Chinese classical music therapy may be an alternative and adjuvant therapy for hypertension, which has important academic value and bright clinical application prospect.

## Data Availability

The raw data supporting the conclusion of this article will be made available by the authors, without undue reservation.

## Abbreviations

BP, blood pressure; SHR, spontaneously hypertensive rats; WN, white noise; WM, Western classical music; CCM, Chinese classical music; RM, rock music.
